# Insights into the biology and insecticide susceptibility of the secondary malaria vector *Anopheles parensis* in an area with long-term use of insecticide-treated nets in northwestern Tanzania

**DOI:** 10.1186/s13071-024-06634-6

**Published:** 2024-12-30

**Authors:** Salum Abdallah Mapua, Ismail Hassan Nambunga, Joel Ouma Odero, Gustav Mkandawile, John Paliga Masalu, Najat Feruz Kahamba, Emmanuel Elirehema Hape, Nancy Stephen Matowo, Frederic Tripet, Fredros Oketch Okumu

**Affiliations:** 1 Environmental Health and Ecological Sciences Department, Ifakara Health Institute, P.O. Box 53, Morogoro, Tanzania; 2 Centre for Applied Entomology and Parasitology, School of Life Sciences, Keele University, Huxley Building, Keele, Staffordshire ST5 5BG UK; 3 School of Biodiversity, One Health and Veterinary Medicine, University of Glasgow, Glasgow, G61 1QH UK; 4 School of Public Health, Faculty of Health Sciences, University of the Witwatersrand, Johannesburg, South Africa; 5 Department of Disease Control, London School of Hygiene and Tropical Medicine, London, UK; 6 Swiss Tropical and Public Health Institute, Kreuzgasse 2, 4123 Allschwil, Switzerland; 7 University of Basel, Basel, Switzerland; 8 School of Life Science and Bioengineering, The Nelson Mandela African Institution of Science and Technology, P.O. Box 447, Arusha, Tanzania

**Keywords:** *Anopheles parensis*, *Plasmodium* spp, Malaria, Tanzania

## Abstract

**Background:**

The *Anopheles funestus* group includes at least 11 sibling species, with *Anopheles funestus* Giles being the most studied and significant malaria vector. Other species, like *Anopheles parensis*, are understudied despite their potential role in transmission. This article provides insights into the biology and insecticide susceptibility of *An. parensis*, with observations of its densities in northwestern Tanzania.

**Methods:**

Mosquitoes were collected in three villages in Misungwi district, northwestern Tanzania, using CDC light traps and battery-powered aspirators indoors and human-baited double net traps outdoors. Female *Anopheles* adults were morphologically sorted and identified by PCR, and a subset was tested by ELISA for vertebrate blood meal sources and *Plasmodium* sporozoite infections. Insecticide susceptibility was assessed using the WHO protocol (2nd edition, 2018). Unfed females were dissected to assess parity, gonotrophic status and insemination status, while blood-fed females were monitored for oviposition to estimate egg counts. The prevalence of *An. parensis* was generally < 24% across all sites, except in Ngaya village, where it unexpectedly constituted 84% of PCR-amplified *An. funestus* sensu lato. This species was present in both indoor and outdoor collections, yet the females exclusively fed on non-human vertebrates, with no human blood meals detected. Parity rates were approximately 49% for resting and 46% for host-seeking females, with slightly higher percentages of both parous and inseminated females in the dry season compared to the wet season. Most parous females had oviposited once or twice, with those in the dry season ovipositing significantly more eggs. The average wing length of female *An. parensis* was 2.93 mm, and there was no significant impact of body size on parity, fecundity or insemination. The *An. parensis* mosquitoes were fully susceptible to pyrethroids, carbamates, organophosphates and organochlorides.

**Results:**

The prevalence of *An. parensis* was generally < 24% across all sites, except in Ngaya village, where it unexpectedly constituted 84% of PCR-amplified *An. funestus* sensu lato. This species was present in both indoor and outdoor collections, yet the females exclusively fed on non-human vertebrates, with no human blood meals detected. Parity rates were approximately 49% for resting and 46% for host-seeking females, with slightly higher percentages of both parous and inseminated females in the dry season compared to the wet season. Most parous females had oviposited once or twice, with those in the dry season ovipositing significantly more eggs. The average wing length of female *An. parensis* was 2.93 mm, and there was no significant impact of body size on parity, fecundity or insemination. The *An. parensis* mosquitoes were fully susceptible to pyrethroids, carbamates, organophosphates and organochlorides..

**Conclusion:**

This study offers insights into the behaviours and insecticide susceptibility of *An. parensis*. Primarily feeding on non-human hosts, *An. parensis* is less significant in malaria transmission than more anthropophilic vectors. Unlike the pyrethroid-resistant *An. funestus* sensu stricto, *An. parensis* remains fully susceptible to public health insecticides despite the use of insecticidal bed nets. These findings provide a foundation for future research and may inform control strategies targeting residual malaria transmission involving *An. parensis*.

**Graphical Abstract:**

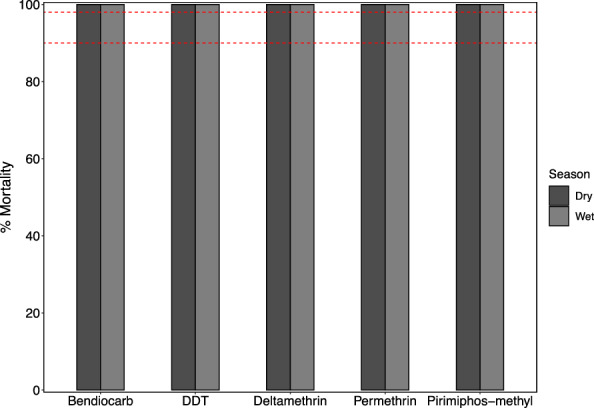

**Supplementary Information:**

The online version contains supplementary material available at 10.1186/s13071-024-06634-6.

## Background

### Findings

Effective malaria control requires understanding key ecological aspects of dominant vector species, including feeding and resting behaviours [1–3], which inform transmission dynamics and vector control strategies. Interventions like insecticide-treated bed nets (ITNs) and indoor residual spraying (IRS) target mosquitoes that bite humans indoors [4–6] but are less effective against species exhibiting behavioural plasticity, such as biting or resting outdoors [7–9]. The *Anopheles gambiae* complex and *An. funestus* group dominate malaria transmission in Africa [10, 11]. *Anopheles funestus* s.s., particularly in East and southern Africa, contributes significantly to transmission and exhibits high insecticide resistance [12–15]. Other members of the *Anopheles funestus* group, including *An. parensis*, *An. rivulorum* and *An. leesoni*, have also been implicated in malaria transmission [16–21]. In Tanzania, *An. parensis* has been reported carrying *Plasmodium falciparum* sporozoites [16, 17, 21], highlighting the need for more research into its role in malaria transmission. Despite findings of malaria-infected sibling species, their biology and response to control interventions remain poorly understood, necessitating further studies on their ecological adaptations and roles in residual transmission. Morphometric traits, such as wing size, are critical indicators of mosquito fitness and ecological adaptations, influencing survival, fecundity and dispersal potential [22–24]. Investigating whether these traits correlate with reproductive parameters, including gonotrophic cycles, fecundity, parity and insemination status, provides insights into the life history and vectorial capacity of *An. parensis*. These analyses are particularly relevant for understanding seasonal and habitat-specific variations that could influence the success of vector control strategies.

**Fig. 1:**
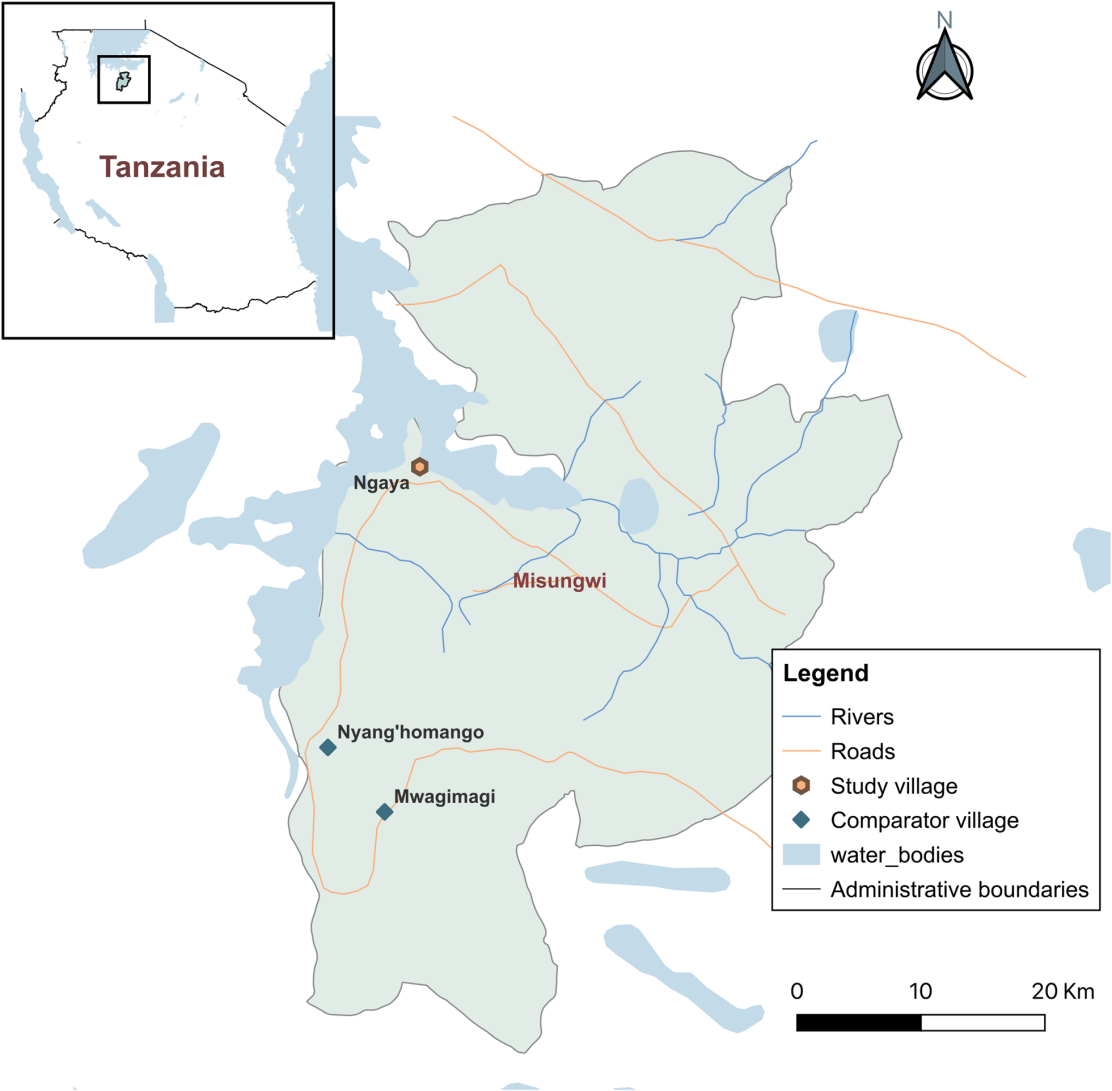
Map showing the study sites in Misungwi district, northwestern Tanzania

We initially set out to investigate the insecticide resistance profiles and genetic structure of *An. funestus* s.s. in different parts of Tanzania, targeting 13 regions with moderate-to-high malaria prevalence [25]. However, during these initial surveys, we noted that while *An. funestus* group from other sites were resistant to pyrethroids, those in one village in Misungwi district were fully susceptible (percentage mortality: 100%). Subsequent genotyping of a 10% subset of the samples used for insecticide susceptibility tests revealed that the mosquitoes were predominantly *An. parensis* rather than *An. funestus* s.s. (Additional file 1). We also detected two individuals infected with *Plasmodium falciparum* sporozoite in *An. parensis* populations from the same district in Tanzania [16]. These findings prompted a series of follow-up surveys, including a comparative survey in other villages (i.e. Mwagimagi and Nyang’homango) in Misungwi district during the first half of 2022 (Fig. 1), which localized the high concentrations of *An. parensis* to Ngaya village in Misungwi district.

Mosquito sampling began in February 2022 but was halted because of early heavy rains. The sampling was resumed and completed in July 2022 (dry season) and January–April 2023 (wet season). Eight households in Ngaya village were selected, and sampling was conducted over 23 days. Resting mosquitoes were collected inside houses using battery-powered Prokopack aspirators (hereafter referred to as Prokopack) from 6 to 8 a.m., while host-seeking mosquitoes were sampled outside the same houses using miniaturized double-net traps (DN-Mini) from 6 p.m. to 6 a.m. Centers for Disease Control and Prevention (CDC) light traps [26] were also used to sample mosquitoes from 6 p.m. to 6 a.m. in the comparator villages exclusively. While the February 2022 and June–July 2022 surveys were done only in Ngaya village, the 2023 sampling was extended to cover three villages, Ngaya, Mwagimagi and Nyang’homango (Fig. 1).

Collected adult mosquitoes were killed by freezing and then sorted morphologically to species level and physiologically as fed, unfed or gravid. Blood-fed females were stored in 1.5-ml centrifuge tubes with 80% ethanol for analysis of blood meal sources and the detection of *P. falciparum* infective sporozoites. Though this study was conducted in a single village with high densities of *An. parensis*, the *An. funestus* s.l. mosquitoes were tested by polymerase chain reaction (PCR) to identify and confirm sibling species [27]. Engorged *An. funestus* mosquitoes were tested using an antibody-sandwich enzyme-linked immunosorbent assay (ELISA) for host blood meal identification [28]. Additionally, a circumsporozoite enzyme-linked immunosorbent assay (CSP-ELISA) was used to detect the presence of *Plasmodium* spp. sporozoites in the mosquito salivary glands [29].

The unfed *An. parensis* females, from both resting and host-seeking collections were dissected to examine their ovaries for parity using the Detinova method [30]. All parous females were further examined for the number of gonotrophic cycles using the Polovodova method [31, 32]. The spermathecae of each female were also dissected and inspected under a microscope to assess insemination status. For fecundity assessment, blood-fed *An. parensis* females were placed individually in paper cups lined with wet filter paper at the bottom to encourage oviposition. A fine mesh was secured over the cups to prevent escape. After oviposition, the number of eggs laid by each female was counted and recorded. In addition to reproductive assessments, the body size of *An. parensis* was evaluated to investigate any correlations between body size and reproductive traits, such as parity, fecundity and insemination status.

Female mosquitoes that were not blood fed were used for baseline insecticide susceptibility bioassays following WHO guidelines [33] with slight modifications as follows: Since we were unable to obtain enough of the required age-synchronized adults collected as larvae, we used adult collected mosquitoes. These mosquitoes were allowed to acclimatize in a local insectary in the study village for at least 8 h to eliminate any moribund or dead individuals before testing; controls were used to monitor excessive mortalities. Insecticides in four different classes were tested at standard WHO recommended doses typically used for major malaria vectors, including: pyrethroid type I (0.75% permethrin), pyrethroid type II (0.05% deltamethrin), carbamate (0.1% bendiocarb), organophosphate (0.25% pirimiphos-methyl) and organochloride (4% DDT). Each test included six replicates, including four with insecticide-impregnated papers and two with oil-impregnated papers as controls. Each replicate contained 20–25 live mosquitoes, totalling a minimum of 120 mosquitoes per assay per candidate insecticide. Mosquitoes were exposed to both insecticide- and oil-impregnated papers for 1 h, with knockdown times recorded at 10, 15, 20, 30, 40, 50 and 60 min. After exposure, mosquitoes were transferred to holding tubes, provided with a 10% glucose solution, and mortality was recorded after 24 h post exposure.

A total of 20,737 mosquitoes were collected indoors and outdoors over 60 trapping nights in three selected study villages. These included 4905 *Anopheles gambiae* s.l., 9474 *An. funestus* s.l., 2489 *An. coustani*, 38 *An. pharoensis*, 26 *An. squamosus*, 110 *An. ziemanni*, 2079 *Culex* spp., 1605 *Mansonia* spp. and 11 *Coquillettidia* spp. The entomological survey results for all study villages are shown in Table 1.

**Table 1:** Mosquito species collected over 60 trap nights in three villages of Misungwi district, northwestern Tanzania

	Ngaya Village		Mwagimagi Village	Nyang’homango Village
Species	Indoor resting collections (Prokopack)	Outdoor host-seeking collections (DN-Mini)	Indoor host-seeking collections (CDC light trap)	Indoor host-seeking collections (CDC light trap)
*Anopheles gambiae* s.l.	702 (19.8%)	1694 (24.3%)	1796 (30.5%)	713 (16.4%)
*An. funestus*	s.l.572 (44.4%)	1676 (24.1%)	3592 (61.1%)	2634 (60.6%)
*An. coustani*	767 (21.6%)	1722 (24.7%)	0 (0%)	0 (0%)
*An. pharoensis*	14 (0.4%)	24 (0.3%)	0 (0%)	0 (0%)
*An. squamosus*	12 (0.3%)	14 (0.2%)	0 (0%)	0 (0%)
*An. ziemanni*	0 (0%)	110 (1.6%)	0 (0%)	0 (0%)
*Culex spp.*	207 (5.8%)	547 (7.9%)	483 (8.2%)	842 (19.4%)
*Mansonia spp.*	269 (7.6%)	1173 (16.8%)	9 (0.2%)	154 (3.5%)
*Coquillettidia spp.*	0 (0%)	8 (0.1%)	0 (0%)	3 (0.1%)
Totals	3,543	6,968	5,880	4,346

Analysis of the *An. funestus* group in all three villages revealed that the dominant species in Ngaya village was *An. parensis*, constituting 84% (1033/1230) of all PCR-amplified samples. In Mwagimagi village, the dominant member of the *An. funestus* group was *An. funestus* s.s., constituting 98.7% of all PCR-amplified samples, while *An. parensis* accounted for only 1.3%. In Nyang'homango village, *An. funestus* s.s. was also the dominant species, making up 75.9% of all PCR-amplified samples. In Nyang'homango, *An. parensis* had a higher proportion compared to Mwagimagi, representing 23.4% of the samples. Subsequent analyses focused on N’gaya village because of the high densities of *An. parensis*. In Ngaya village, 3248 *An. funestus* s.l. females were collected inside and outside homes over 23 days, with the majority (*n* = 1676) from host-seeking catches (Table 1).

A total of 257 out of 298 *An. parensis* mosquitoes from indoor resting collections were analysed, with 93% (*n* = 238) having fed on cattle, 2% (*n* = 6) on dogs and 1% (*n* = 2) on goats (Table 2). Additionally, 177 out of 735 *An. parensis* females collected while host-seeking outdoors were analysed, with most (60%, *n* = 107) feeding on cattle and 2% (*n* = 4) on pigs. Over 30% of the outdoor host-seeking *An. parensis* were non-reactive for all blood sources tested (Table 2). A subset of mosquitoes from resting collections (*n* = 463) and host-seeking collections (*n* = 767) were screened for *Plasmodium* spp., but none tested positive in this round. However, a previous survey had detected two infected samples from this same site [16].

**Table 2:** Sources of blood meals taken by *Anopheles parensis* mosquitoes from the indoor resting and outdoor host-seeking collections

Host type	Indoor collections (Prokopack aspirators)	Outdoor Collections (DN-Mini Traps)
Human	0	0
Cattle	238 (93%)	107 (60%)
Pig	0	4 (2%)
Goat	2 (1%)	0
Dog	6 (2%)	0
Non-reactive	11 (4%)	66 (37%)
Total tested	257	177

**Fig. 2:**
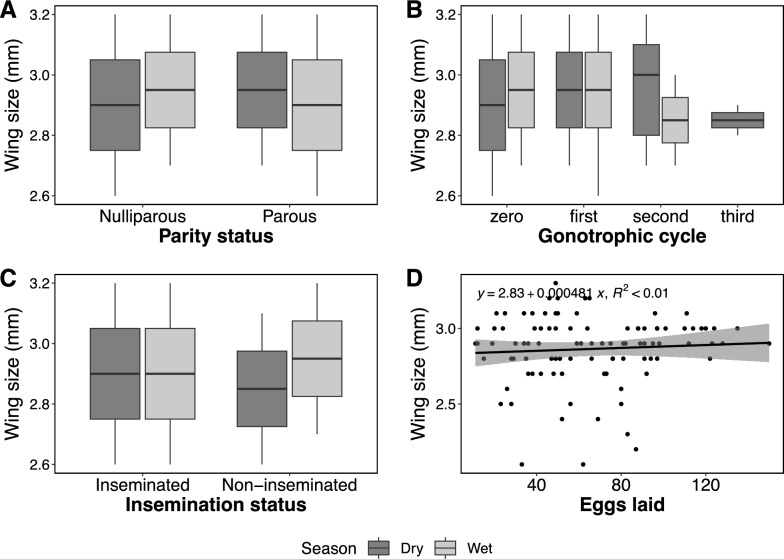
Relationship between wing sizes of wild-caught *Anopheles parensis* and (**A**) parity status, (**B**) gonotrophic cycles, (**C**) insemination status and (**D**) number of eggs oviposited in both wet and dry seasons

**Fig. 3:**
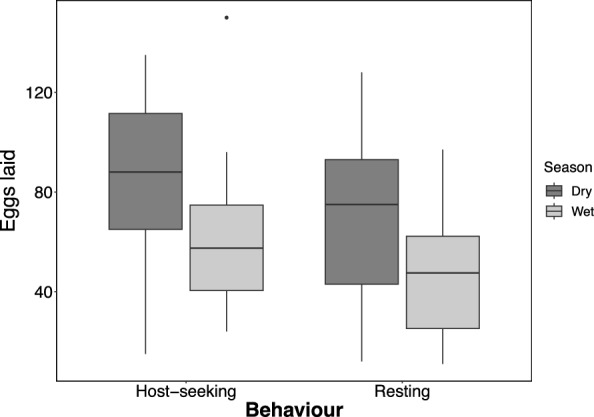
Number of eggs oviposited by wild-caught *Anopheles parensis* from both resting and host-seeking catches (representing mosquitoes initially captured during host-seeking attempts, regardless of their physiological status at the time of collection) in the dry (July) and wet (February) seasons

Overall, 49% of *An. parensis* females collected resting indoors were parous compared to 46% among those collected in double-net traps outdoors. The proportion of parous females was higher during the dry season than in the wet season, although this difference was not statistically significant (Additional file 2). Further examination revealed that most parous females had laid eggs once (85.1%) or twice (10.6%), with none having laid eggs more than three times (Fig. 2). It was also observed that more *An. parensis* were inseminated during the dry season than in the wet season, but this difference was not statistically significant (Additional file 3). Similarly, regarding fecundity, those collected during the dry season laid more eggs (mean = 76.8, 95% CI  [67.6, 86]) than those collected during the wet season (*p* < 0.001). Moreover, *An. parensis* collected outdoors in double net traps laid significantly more eggs (mean = 72.5, 95% CI [63.6, 81.5]) than those collected resting indoors (*p* < 0.001) (Fig. 3).

The mean wing size for *An. parensis* females was 2.93 mm (95% CI [2.91, 2.95]), slightly larger than the 2.65 mm (95% CI [2.42, 2.89]) observed in *An. funestus* s.s. mosquitoes in the same district (Odero et al., unpublished data). There was no statistically significant difference in wing sizes related to gonotrophic cycles, fecundity, parity or insemination status (Fig. 2).

The *An. parensis* populations were fully susceptible to all tested insecticides, showing 100% mortality at the discriminating concentrations (Additional file 4). Knockdown times varied among the insecticides, reflecting their modes of action. Pyrethroids, known for their fast-acting neurotoxic effects on sodium channels, achieved a 50% knockdown (KDT_50_) more quickly compared to other insecticides (Table 3) in both dry and wet seasons. Similarly, they reached KDT_95_ in less time during the wet season compared to the dry season. DDT exhibited a longer KDT_50_ compared to pyrethroids, consistent with its slower action through prolonged excitation of sodium channels. Additionally, Table 3 indicates that pirimiphos-methyl had longer KDT_50_ and KDT_95_ during the dry season compared to the wet season, while the opposite was true for bendiocarb. The variations in KDT times underscore the functional differences between fast-acting (i.e. pyrethroids) and slower-acting (i.e. DDT, pirimiphos-methyl) insecticides, highlighting the influence of the insecticide's mode of action on knockdown efficacy. Although pyrethroids achieved a KDT_50_ in < 2 min during the dry season, it took > 30 min to reach KDT_95_ (Table 3). These seasonal differences in KDT_50_ and KDT_95_ may reflect changes in mosquito physiology or behaviour during the wet and dry seasons, potentially influencing their susceptibility to insecticides.

**Table 3:** Knockdown times of *Anopheles parensis* mosquitoes at discriminating concentrations in tests done in dry and wet seasons in Ngaya, northwestern Tanzania

Season	Insecticide	Class	Dose	KDT_50_ ± SE	KDT_95_ ± SE
Dry season	Bendiocarb	Carbamate	0.1%	23.1 ± 5.2	32.9 ± 10.1
	DDT	Organochlorine	4%	21.1 ± 5.3	32.1 ± 11.0
	Deltamethrin	Pyrethroid	0.05%	−5.0 ± 42.5	41.5 ± 31.1
	Permethrin	Pyrethroid	0.75%	1.8 ± 26.5	33.1 ± 22.4
	Pirimiphos-methyl	Organophosphate	0.25%	34.9 ± 8.5	57.3 ± 16.8
Wet season	Bendiocarb	Carbamate	0.1%	24.9 ± 5.6	35.7 ± 10.6
	DDT	Organochlorine	4%	33.0 ± 8.3	54.9 ± 16.3
	Deltamethrin	Pyrethroid	0.05%	10.0 ± 5.1	17.1 ± 7.4
	Permethrin	Pyrethroid	0.75%	6.3 ± 14.5	18.8 ± 11.9
	Pirimiphos-methyl	Organophosphate	0.25%	27.4 ± 6.3	40.6 ± 11.7

Our findings provide important insights into the biology, behaviour and insecticide susceptibility of *An. parensis* in Tanzania, which may also be relevant to other regions. While *An. parensis* rests indoors, its females primarily feed outdoors on non-human hosts, indicating a limited role in malaria transmission in the absence of major vectors. High densities observed in outdoor host-seeking collections suggest a preference for outdoor biting, warranting further investigation into indoor-outdoor interactions. Although rarely found carrying *P. falciparum* sporozoites, the prevalence of *An. parensis* highlights the need for broader control strategies, especially since it remains fully susceptible to public health insecticides and has significant indoor resting populations. This raises questions about its survival despite the widespread use of insecticide-treated nets (ITNs) in the area.

Most *An. parensis* were found to feed on cattle, with some also feeding on dogs, pigs and goats, but none on humans, reflecting its zoophilic nature [34]. However, our findings suggest that *An. parensis* can opportunistically feed on both humans and animals depending on availability. Previous findings indicate that *Plasmodium*-infected *An. parensis* have been observed [16, 17, 35], supporting the notion of occasional human bites. In Ngaya village, *An. parensis* populations were fully susceptible to common insecticides, unlike the resistant *An. funestus* s.s. in Tanzania [25]. Despite this susceptibility, *An. parensis* persists in high densities in areas with widespread dual-active ITNs, raising questions about its survival indoors despite effective insecticide coverage. One possible explanation could be its behavioural tendencies, such as outdoor resting or feeding, which might reduce exposure to insecticides. Alternatively, differences in insecticide pressure or genetic factors may play a role in maintaining susceptibility. These aspects merit further investigation.Additionally, there was no significant difference in parity or insemination status of *An. parensis* between resting and host-seeking catches across seasons. However, more parous females were found in the dry season, indicating the species’ ability to endure dry conditions, similar to *An. funestus* [36]. Wing size did not vary with gonotrophic cycles, insemination status or parity. Notably, *An. parensis* from the dry season laid more eggs, suggesting seasonal differences in gonotrophic cycles [37, 38], possibly because of environmental stress prompting multiple blood meals per cycle [37–39].

Overall, this study underscores the biology and behaviour of *An. parensis* as a minor malaria vector in Tanzania. In the Misungwi district, it was the predominant species within the *An. funestus* group, even though malaria transmission is primarily driven by major vectors like *An. funestus* s.s. The species' preference for outdoor biting and non-human hosts limits its transmission potential. Nevertheless, its full susceptibility to common insecticides suggests that integrating indoor residual spraying (IRS) with ITNs could be an effective control strategy, especially given its indoor resting behaviour. Although *An. parensis* predominantly feeds on cattle, its occasional feeding on humans could facilitate the transfer of *P. falciparum*, suggesting its potential role as a bridge vector. These findings lay a solid foundation for future research and control strategies.

## Supplementary Information

Supplementary material 1. Additional file 1: Proportion of sibling species within the *Anopheles funestus* group across various districts in mainland Tanzania; Additional file 2: Analysis of parity status in *Anopheles parensis*; Additional file 3: Multivariate analysis of insemination in *Anopheles parensis*; Additional file 4: Percentage mortality of *Anopheles parensis* mosquitoes exposed to discriminating concentrations of candidate insecticides. The red-dotted lines indicate 90% and 98% mortality thresholds, marking resistance and susceptibility.

## Data Availability

All data supporting the conclusions of this article are provided within the text and supplementary material.

## Abbreviations

ITN: Insecticide-treated bed net
IRS: Indoor residual spraying
PCR: Polymerase chain reaction
CDC: Centers for Disease Control and Prevention
ELISA: Enzyme-linked immunosorbent assay
CSP-ELISA: Circumsporozoite enzyme-linked immunosorbent assay
WHO: World Health Organization
DDT: Dichlorodiphenyltrichloroethane
CI: Confidence interval
KDT: Knock-down time
DN-mini: Miniaturized double net
