# Supplementary material for: Insights into the biology and insecticide susceptibility of the secondary malaria vector Anopheles parensis in an area with long-term use of insecticide-treated nets in northwestern Tanzania
Source: Parasit Vectors. 2024 Dec 30;17:549. doi: 10.1186/s13071-024-06634-6 (PMC11687015; doi:10.1186/s13071-024-06634-6)
Supplement: Supplementary file 1 — Supplementary material 1. Additional file 1: Proportion of sibling species within the Anopheles funestus group across various districts in mainland Tanzania; Additional file 2: Analysis of parity status in Anopheles parensis; Additional file 3: Multivariate analysis of insemination in Anopheles parensis; Additional file 4: Percentage mortality of Anopheles parensis mosquitoes exposed to discriminating concentrations of candidate insecticides. The red-dotted lines indicate 90% and 98% mortality thresholds, marking resistance and susceptibility. [file 13071_2024_6634_MOESM1_ESM.docx]

**Additional file 1:** Proportion of sibling species within the *Anopheles funestus* group across various districts in mainland Tanzania (Odero *et al*., Unpublished data).

**Additional file 2:** Analysis of parity status in *Anopheles parensis*

| Variable | Total (N) | Parous n (%) | OR (95% LC-UC) | p-value |
| --- | --- | --- | --- | --- |
| Resting | 100 | 49 (49) | 1 | 0.71 |
| Host-seeking | 100 | 46 (46) | 0.10 (0.51-1.58) |  |
| Dry | 100 | 55 (55) | 1 | 0.08 |
| Wet | 100 | 41 (41) | 0.60 (0.34-1.05) |  |

*Percentage parous = parous (n)/Total number of mosquitoes examined (N)

**Additional file 3:** Multivariate analysis of insemination in *Anopheles parensis*

| Variable | Total (N) | Insemination n (%) | OR (95% LC-UC) | p-value |
| --- | --- | --- | --- | --- |
| Resting | 100 | 67 (67) | 1 | 0.58 |
| Host-seeking | 100 | 70 (70) | 1.19 (0.65-2.17) |  |
| Dry | 100 | 73 (73) | 1 | 0.20 |
| Wet | 100 | 64 (64) | 0.68 (0.37-1.24) |  |

*Percentage insemination = insemination (n)/Total number of mosquitoes examined (N)

**Additional file 4:** Percentage mortality of *Anopheles parensis* mosquitoes exposed to discriminating concentrations of candidate insecticides. The red-dotted lines indicate 90% and 98% mortality thresholds, marking resistance and susceptibility.
